# Angiogenic inhibitors delivered by the type III secretion system of tumor-targeting *Salmonella typhimurium* safely shrink tumors in mice

**DOI:** 10.1186/s13568-016-0226-8

**Published:** 2016-08-24

**Authors:** Lei Shi, Bin Yu, Chun-Hui Cai, Jian-Dong Huang

**Affiliations:** 1 Faculty of Medicine, School of Biomedical Sciences, The University of Hong Kong, L3-72, Laboratory Block, 21 Sassoon Road, Pokfulam, Hong Kong; 2 Shenzhen Institute of Research and Innovation, The University of Hong Kong, Shenzhen, People’s Republic of China; 3 Key Laboratory of Optoelectronic Devices and Systems of Ministry of Education and Guangdong Province, College of Optoelectronic Engineering, Shenzhen University, Shenzhen, People’s Republic of China; 4 Department of Obstetrics and Gynaecology, The University of Hong Kong, Pokfulam, Hong Kong; 5 Advanced Institute of Translational Medicine, Tongji University School of Medicine, Shanghai, People’s Republic of China; 6 The Centre for Synthetic Biology Engineering Research, Shenzhen Institutes of Advanced Technology, Shenzhen, People’s Republic of China

**Keywords:** Bacterial cancer therapy, Tumor-targeting, *Salmonella typhimurium*, Viable region, Type III secretion system, Anti-angiogenesis

## Abstract

Despite of a growing number of bacterial species that apparently exhibit intrinsic tumor-targeting properties, no bacterium is able to inhibit tumor growth completely in the immunocompetent hosts, due to its poor dissemination inside the tumors. Oxygen and inflammatory reaction form two barriers and restrain the spread of the bacteria inside the tumors. Here, we engineered a *Salmonella typhimurium* strain named ST8 which is safe and has limited ability to spread beyond the anaerobic regions of tumors. When injected systemically to tumor-bearing immunocompetent mice, ST8 accumulated in tumors at levels at least 100-fold greater than parental obligate anaerobic strain ST4. ST8/pSEndo harboring therapeutic plasmids encoding Endostatin fused with a secreted protein SopA could target vasculature at the tumor periphery, can stably maintain and safely deliver a therapeutic vector, release angiogenic inhibitors through a type III secretion system (T3SS) to interfere with the pro-angiogenic action of growth factors in tumors. Mice with murine CT26 colon cancer that had been injected with ST8/pSEndo showed efficient tumor suppression by inducing more severe necrosis and inhibiting blooding vessel density within tumors. Our findings provide a therapeutic platform for indirectly acting therapeutic strategies such as anti-angiogenesis and immune therapy.

**Electronic supplementary material:**

The online version of this article (doi:10.1186/s13568-016-0226-8) contains supplementary material, which is available to authorized users.

## Introduction

Previous studies of tumor have mostly focused on discovering the genetic and epigenetic abnormality of cancer cells, which were used to explore therapeutic strategies against solid tumors (Kopnin 2000). During recent years, there has been intense interest in understanding the unique tumor microenvironment: hypoxia (Ke et al. 2012), angiogenesis (Yamato et al. 2012), heterogeneous cellular interaction (Leonardi et al. 2012) and immunosuppressive environment (Shiao et al. 2011). These characteristics hamper the intratumoral delivery of therapeutic molecules and favor the development of treatment-resistant cells. Progresses in this field have enabled us to control the growth and metastasis of the solid tumor and develop new cancer treatment options.

Recently, more and more studies nicely show that some anaerobic bacteria can accumulate in tumor hypoxia regions after systemic infection and be used as tumor specific therapeutic agents in cancer therapy (Bernardes et al. 2013; Hong et al. 2014; Moreno et al. 2010). Hypoxia resulted from irregular vascular network and immune suppression in the tumor microenvironment allowed these bacteria to replicate, accumulate and elicit locally restricted cytotoxic effects leading to a continuous tumor regression. For instance, facultative anaerobic *Salmonella* contains lipolysaccharide and virulent factors which kill cancer cells directly and strongly trigger host immune system to destruct tumors (Fordham et al. 2012; Lee 2012; Yoon et al. 2011). Additionally, metabolically active bacteria can express a substantial amount of therapeutic molecules and continuously release them during infection. It leads to a high concentration of anti-cancer factors accumulated inside the solid tumors. These indicated that engineered bacteria are well suited for incorporating multiple targets into a single system. A growing number of studies have reported the successful targeting and treatment of murine tumors by bacteria. Researchers found that the bacterial treatment to be effective only against relatively small tumors in immunocompromised nude mice; a complete regression of established tumors in immunocompetent hosts has been described rarely thus far (Friedlos et al. 2008; Hu et al. 2009; Nagakura et al. 2009).

No selected bacterium that is able to inhibit tumor growth completely, mainly by two reasons: (1) inflammatory reaction (mainly by neutrophils) and relatively high oxygen levels at the viable rim restrain the spread of obligate anaerobes; and (2) the outer layer of tumor tissue was unaffected, in which the tumor cells rapidly proliferate and invade surrounding tissues with the support of tumor vasculatures. Here, we confer *Salmonella* with limited ability to spread beyond the anaerobic regions of tumors to target blood vessels with bacterial expressed an angiogenic inhibitor, Endostatin, which are secreted through a type III secretion system (T3SS) to interfere with the pro-angiogenic action of growth factors (O’Reilly et al. 1997). We used the engineered *S. typhimurium* strain ST8 to deliver Endostatin fused with a type III secretion protein SopA in order to efficiently secrete by bacteria and diffuse through tumor tissue. The ability to diffuse through the tissues will augment therapeutic effects, and enable repression of blood vessels in the cancer cell proliferating regions.

## Materials and methods

### Bacterial strains and plasmid construction

*Escherichia coli* strain DH5α (Invitrogen) was used for cloning experiments. *Salmonella typhimurium* strain SL7207 stain has the following genotype: *S.* t*yphimurium* 2337-65 derivative hisG46, DEL407 [aroA::Tn10 (Tcs)] was obtained from Dr. B.A.D. Stocker (1981). Plasmid pSim6 was a gift from Dr. D.L. Court (2006). All bacterial strains used in this study are listed in Additional file 1: Table S1. Primers are listed in Additional file 1: Table S2. All *S. typhimurium*n strains were grown in Luria broth (LB) containing 0.3 M sodium chloride to activate the expression of the T3SS.

### Mammalian cell culture

Mouse colon cancer CT26 cell line was obtained from ATCC, and routinely cultured under conditions specified by the manufacturer. Human umbilical vein endothelial cells (HUVEC) were obtained from Dr. E.H.C. Tang from the Department of Pharmacology & Pharmacy, at the University of Hong Kong.

### Western blot analysis

Condensed medium or homogenized tissues were collected and lysed. The heat-denatured samples were loaded and separated by SDS-PAGE gel. When the electrophoresis was ended, the protein samples in the gel were transferred onto PVDF membranes (Roche). Membranes were blocked in 5 % non-fat milk in Tris-buffer saline with 0.1 % Tween-20, and further incubated with primary and secondary antibodies to detect the presence of different proteins and visualized by chemiluminescence detection kit (Pierce). Antibodies against Flag (Sigma-Aldrich) and α-tubulin (Cell signaling technology) were used. Corresponding horseradish peroxidase-conjugated secondary antibodies were purchased from Invitrogen.

### Bacterial distribution and plasmid stability test in vivo

After systemic administration, mice were killed and different organs were removed and weighted. Tissues were homogenized in 9 volumes of H_2_O and colony forming units (CFU) test of viable *Salmonella* were determined by plating serial dilutions on LB agar plates supplemented with streptomycin or the antibiotic corresponding to the construction plasmid as well as diaminopimelic acid (DAP) (Sigma-Aldrich).

### Establishment of tumor xenografts and evaluation of therapeutic effects

BALB/c mice were obtained from the Laboratory Animal Unit of University of Hong Kong. 10^5^ CT26 murine colon cancer cells were injected to the inguinal mammary fat pads of female mice (6–8 weeks of age). When the tumors reached ~400 mm^3^ in size, the mice were received treatments. All animal procedures were approved by the Ethics Committee of University of Hong Kong and done according to institutional guidelines.

Bacteria were injected through the tail vein (5 × 10^7^ CFU/100 μl PBS). In control animals, PBS was injected in the same volume. Mice were examined and the tumor diameters were measured every other day in two dimensions with an external microcaliper. Subcutaneous tumor size was calculated by using the formula:

$${\text{Tumor}}\;{\text{volume}}\; = \; {\text{length}}\; \times \; {\text{width}}^{ 2} \; \times \;0. 5 2.$$

### Histology and immunohistochemistry

Tumor samples and the normal organs from therapy studies were fixed immediately with 4 % paraformaldehyde. After incubation, the samples were washed and dehydrated in graded ethanol. After appropriate permeation in xylene, the fixed tissues were embedded in paraffin and followed by cutting 7 μm paraffin sections. Then they were de-paraffinised xylene twice and rehydrated in descending concentration of ethanol. Standard hematoxylin–eosin (H&E) staining of paraffin embedded tissue was used for histological examination.

The sections along the H&E stained slides were further processed for immunohistochemisty. Heat-induced antigen retrieval was performed at temperature >95 °C in 10 mM sodium citrate buffer (pH6.0) and followed by cooling down at room temperature. Endogenous peroxidase activity was quenched by incubating with 3 % hydrogen peroxide for 10 min. Then the background staining was blocked by incubation with blocking buffer (0.1 % Triton X-100, 3 % BSA and 2 % normal donkey serum) for 1 h. These experiments were carried out using the following first antibodies: anti-*Salmonella* (Abcam), anti-Flag (Sigma-Aldrich), anti-CD105 (BD PharMingen) and anti-Ki67 (Sigma-Aldrich). After washing with PBS, horseradish peroxidase-conjugated secondary antibodies were applied. Finally, the sections were stained with a freshly prepared 3,3′-diaminobenzidine (DAB) chromogen using a DAB kit (DAKO) and then counterstained with hematoxylin. Photos were taken under Olympus BX51 microscope by using a bright-field illumination.

### Determination of hemoglobin content in tumors

Tumors were weighted, homogenized in PBS buffer and centrifuged; the content of hemoglobin in the supernatant was analyzed by Drabkin’s reagent (Sigma-Aldrich) and normalized to the weighs.

### Statistical analysis

All statistical analysis was performed with Prism software (GraphPad Prism). Statistical comparisons between two groups were evaluated by student’s *t* test. All the data are represented as mean plus standard deviation (SD). The differences were considered statistically significant when *P* value was less than 0.05.

## Results

### Development and characterization of tumor-targeting *Salmonella* strain ST8

**Fig. 1:**
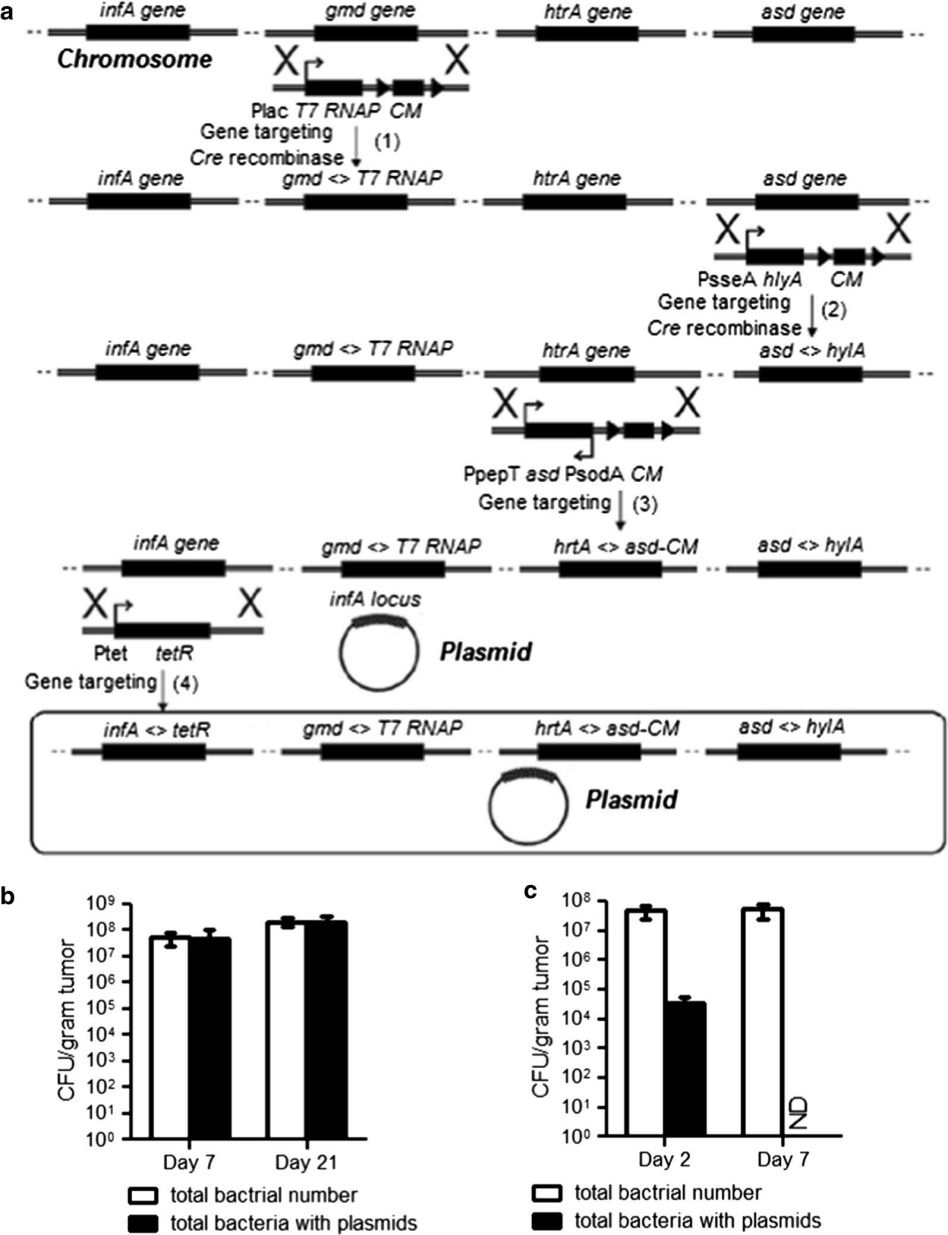
Generation of a tumor-targeting *Salmonella* strain ST8 for delivery and expression of multiple therapeutic factors. **a** Schematic diagram of the creation process of strain ST8. **b** CFU tests of ST8/pcDNA-infA inside tumors of nude mice following intravenous injection. Tumors were collected at 7 and 20 days after bacterial infection, homogenized in PBS and plated onto agar plates with or without ampicillin selection to determine the counts of recombinant and total bacteria, respectively. Values are expressed as mean ± SD, n = 5. **c** SL7207/pcDNA-infA infected tumors were homogenized and analyzed for the total bacterial number and total bacteria with the plasmids. ND stands for CFU = 0. Shown is the mean CFU per gram tissues plus SD

Using enhanced recombineering methods, we previously developed an “obligate” anaerobic *Salmonella* strain YB1 and ST4 with enhanced host safety and anti-tumor activity (Guo et al. 2015; Shi et al. 2016; Yu et al. 2012). However, early metastases and viable tumor cells outside necrotic regions are well or partially oxygenated, which are inaccessible to obligate anaerobic bacteria. To increase the fitness in the non-hypoxic, outer rim of the solid tumor and metastases, replication-competent strains ST7 and ST8 have been developed (Fig. 1a; Additional file 1: Table S1). Strain ST8 is a derivative of *S. typhimurium* ST4 strain (Shi et al. 2016), which has the following genotype: *S. typhimurium* 2337-65 derivative hisG46, *△aroA::Tn10 (Tcs), △gmd::Plac*-*T7RNAP, △htrA::PpepT*-*asd*-*PsodA,△infA::Ptet*-*tetR*. Chloramphenicol resistance gene in ST4 was eliminated by induction of *Cre* recombinase. Once the *loxp* sites have been removed, the transcription of *asd* gene is controlled by the upstream *htrA* promoter, which resulted in a very leaky expression under normal oxygen levels. This mutant can penetrate into tumor tissue and effectively colonize viable regions of tumors otherwise unaffected by standard cancer therapy and express multiple therapeutic molecules.

Maintaining sustained levels of angiogenesis inhibitors is crucial for prolonged suppression of angiogenesis. The stability and copy number of therapeutic plasmid in bacterial hosts also significantly affect the expression level of target gene. To solve this problem, a small essential gene *infA* from *E. coli* (encoding for translation initiation factor 1, IF1) was inserted into the exogenous plasmid which has been deleted from the chromosome. As a consequence, only plasmid-carrying bacterial cells can survive, making this strain totally dependent on the maintenance of the *infA*^+^ plasmid. We investigate wherever the exogenous plasmid pcDNA-infA (pcDNA derivative with *E.coli infA* locus) were stably present in vivo by comparing the total bacterial number with the amount of plasmid-carrying cells in murine tissue. After 7and 21 days, ST8/pcDNA-infA isolated from the tumors and plated onto agar plates with or without antibiotic selection. No statistic difference of colony forming units (CFU) was detected in total and recombinant bacteria carrying plasmids (Fig. 1b), indicating that the presence of exogenous plasmids in the bacteria did not compromise the growth of the bacteria and the plasmids were stably maintained in the bacteria over 3 weeks. In comparison, 100 % parental strain SL7207 had lost the plasmids at day 7 (Fig. 1c).

**Fig. 2:**
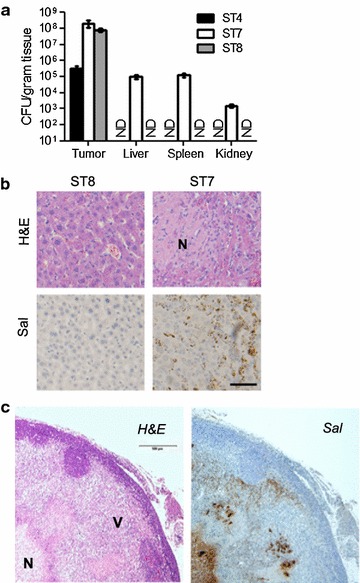
The conversion of obligate anaerobic strain ST4 to ST8 could target the viable regions inside solid tumor and prevent the bacterial killing of the mice. **a** Distribution and tumor colonization of ST4, ST7 and ST8 in tumor-bearing nude mice. Different organs were collected at 14 days after bacterial infection and analyzed for the presence of bacteria (ND stands for CFU = 0). Shown is the mean CFU per gram tissues plus SD. **b** Representative histopathologic and immunohistochemical staining of *Salmonella* on liver sections as indicated. *Scale bar* 100 μm. Necrotic region was marked by *N*. **c** Representative histopathologic and immunohistochemical detection of *Salmonella* identified macroscopic regions of the viable region (marked with *V*), necrotic area (marked with *N*) and bacterial colonization. *Scale bar* 500 μm

In addition, the bacterial distribution tests of ST4, ST7 or ST8 strain in animals within the implanted tumors were investigated. Three groups of immunocompetent BALB/c mice were inoculated with CT26 colon cancer cells and when tumor volumes reached 300–500 mm^3^, a single dose (5 × 10^7^) of bacteria was injected intravenously. Bacterial distribution tests in the immunocompetent mice have shown that systemically injected ST8 and control strain ST7 (without hypoxic control circuit), a large number of bacteria accumulate within the solid tumors, achieving about 10^8^–10^9^ cfu/gram tissues at day 14. Because of a high degree of bacterial accumulation within tumors, these high titers can enhance the therapeutic effects, as the high amount therapeutic molecules generated and delivered by bacteria. In contrast, the numbers of obligate anaerobes ST4 in tumors were only 10^5^ cfu/gram (Fig. 2a). For ST7 inoculated mice, 10^3^–10^5^ CFU/gram of bacteria were found in normal organs. The unspecific accumulation of ST7 in livers caused hepatic injury (Fig. 2b). In comparison, tuning survival gene *asd* expression cassette, ST8 was totally cleared from all the organs except the heavily immune-suppressed microenvironment of metastases and primary tumor by 3 weeks (Additional file 1: Figure S1). ST8 treated tumors exhibited huge necrotic regions and a small viable rim surrounding the severe necrosis. Figure 2c shows a high magnification of a H&E-stained paraffin section of the ST8 infected tumors. The viable parts (V) of the tumor were stained purple, whereas the necrotic areas (N) of the tumor appear white. A more detailed examination of the distribution of ST8 inside the tumors revealed that it could target the outer rim and predominantly, although not exclusively, resided in the hypoxic/necrotic regions of the tumors. Controlled growth of ST8 could prevent a risk for sepsis in the clinical setting.

### Endostatin-expressing ST8/pSEndo inhibits angiogenesis in vitro

**Fig. 3:**
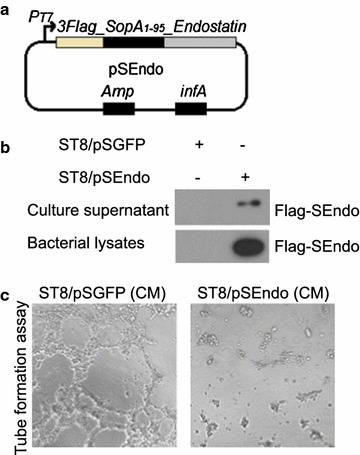
ST8/pSEndo secretes recombinant SopA_Endostatin to target cells through the type III secretion system. **a** Diagram of plasmid pSEndo used in this study. **b** Plasmid pSGFP and pSEndo were introduced into ST8. ST8/pSEndo cells were exposed to cultured CT26 cells and the presence of fusion protein in supernatants examined as described above. **c** Endothelial cell tube formation assay showed interference of network assembly of HUVEC cells on Matrigel in ST8/pSEndo conditioned medium (*right*) compared with ST8/pSGFP conditioned medium (*left*)

*Salmonella typhimurium* possesses a T3SS, a macromolecular, needle-like apparatus necessary for infection by secreted proteins (Galan and Wolf-Watz 2006). In this report, we modified *Salmonella* to bear a plasmid vector allowing for stable expression of angiogenic inhibitors. To endow the targeted *S. typhimurium* strain for secreting endostatin, we used the plasmid pSEndo, in which a mouse *Endostatin* cDNA was fused with a N-terminal FLAG epitope tag and a *SopA* N-terminal sequence (the first 95 residues of SopA) which directs protein secretion through T3SS (Fig. 3a). The activation of T3SS is stimulated upon contact with eukaryotic cells. Hence, we exposed the ST8/pSEndo cultured epithelial CT26 cells at a multiplicity of infection (MOI) of 200 for 6 h and recovered culture supernatant. The level of the fusion protein was assessed by western blot analysis of condensed culture medium (Fig. 3b).

We next studied whether the endostatin fusion proteins secreted by ST8/pSEndo has direct anti-angiogenic effect on human endothelial cells. Conditioned medium (CM) was harvested from CT26 cells’ culture medium after 6 h exposure with ST8/pSEndo or ST8/pSGFP (mock control). ST8/pSEndo conditioned medium significantly inhibited the ability of HUVEC to form tube-like structures in a matrigel angiogenesis assay, whereas ST8/pSGFP conditioned medium had no such effect (Fig. 3c). These data suggested that ST8/pSEndo could release biologically active angiogenic inhibitors into the medium and inhibit in vitro angiogenesis, which encouraged us to determine whether tumor inhibition by ST8/pSEndo could be realized in animal models.

### Delivery of endostatin using ST8/pSEndo inhibits tumor angiogenesis

**Fig. 4:**
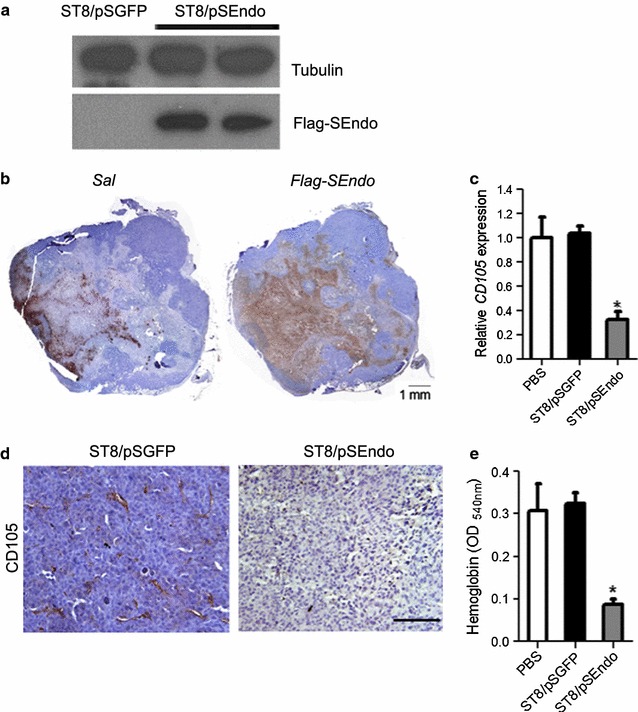
Injection of ST8/pSEndo significantly retards tumor angiogenesis. Detection of Flag-SEndo expression in the ST8/pSEndo treated tumors by western blot analysis (**a**) and immunohistochemical assay (**b**). **b** Composite images of a whole tumor infected with ST8/pSEndo stained to visualize bacteria (*left*) and Flag-SEndo (*right*). Immunohistochemical assay on tumor sections revealed that the therapeutic polypeptide diffused around the bacteria and some molecules have been found to be transferred to the outer rim. Quantitative RT-PCR analysis (**c**) and immunohistochemistry (**d)** to detect CD105-positive endothelial cells in tissue sections from ST8/pSGFP or ST8/pSEndo treated tumors. *Scale bar* 100 μm. **e** Angiogenesis was analyzed by quantification of haemoglobin content (n = 3). *P < 0.05

To assess in vivo effects, mice bearing established subcutaneous flank CT26 tumors were intravenously injected with ST8/pSEndo or ST8/pSGFP. After 14 days, the mice were sacrificed, and then tumors were excised. In animals that received ST8/pSEndo, tumor-specific expression of endostatin fusion protein was confirmed by western blot analysis (Fig. 4a). To study the bacterial colonization and distribution of endostatin inside the tumors, immunohistochemistry assays on tumor sections were carried out. As shown in Fig. 4b, *Salmonella* ST8 secretion of angiogenic inhibitors formed a spatial diffusion pattern throughout tumor tissues and target both necrotic and proliferative tumor regions, indicating that ST8/pSEndo successfully presents intratumoral pools of angiogenic inhibitors that diffused into surrounding tissue after colonization of tumors.

The endothelium in primary tumors was analyzed by detection of activated endothelial cell marker endoglin (CD105), which vascular expression is limited to proliferating cells. Relative level of CD105 transcripts in ST8/pSEndo treated tumors were 68.9 % (P = 0.0002) lower than those in mice injected with vector control ST8/pSGFP (Fig. 4c). Imaging of the blood vessels by immunohistology indicated that continuous release of endostain fusion proteins within tumors successfully suppressed angiogenesis in comparison to the control group treated with ST8/pSGFP (Fig. 4d). The vessel densities correlated with the mean hemoglobin content, with the ST8/pSEndo treated animals showing significantly less hemoglobin in tumors as compared to controls (P = 0.0012) (Fig. 4e). Devascularization caused by bacterial-mediated anti-angiogenic therapy increases tumor hypoxia, and that this hypoxia accelerated the growth of bacteria which further augments the effect of loss of vascular support on the tumors.

### Potent antitumor activities in experimental colon carcinoma by S*almonella* mediated expression of angiogenic inhibitor endostatin

**Fig. 5:**
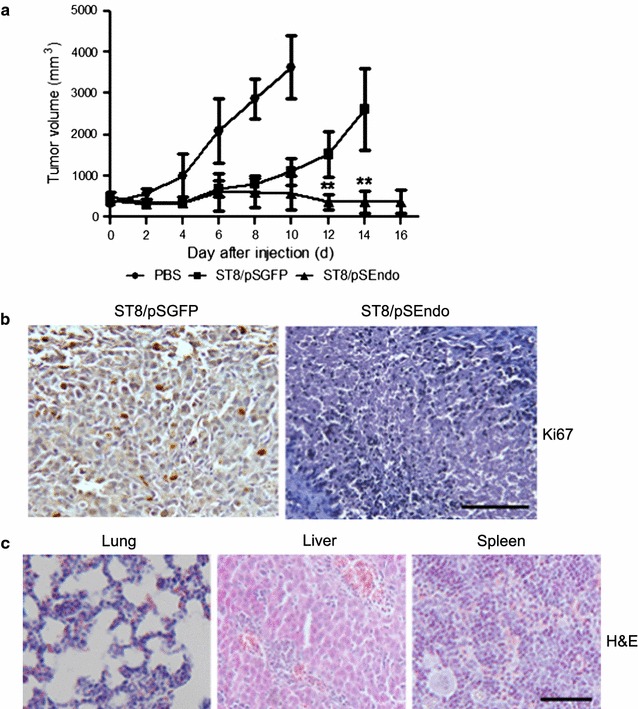
Therapeutic effects in the immunocompetent BALB/c mice with highly aggressive tumors elicited by ST8-mediated secretion of Endostatin. **a** Potent treatment of large established tumors with ST8/pSEndo. CT26 murine tumor cells were implanted subcutaneously into BALB/c mice, and then the mice bearing large tumors received intravenous injections of the indicated strains. Values are expressed as mean ± SD (n = 5). **P < 0.01 compared with vector control ST8/pSGFP. **b** Tumor proliferation was examined by Ki67 expression of primary tumors from ST8/pSGFP and ST8/pSEndo treated mice. *Scale bar* 100 μm. **c** No apparent damages were found in any of the organs in the ST8/pSEndo treatment group. *Scale bar *100 μm

Most engineered bacteria were found to effective only against relatively small tumors in studies (Chen et al. 2009; Hong et al. 2014; Zhao et al. 2012), we tested the therapeutic effects of ST8/pSEndo on large tumors (initial size of 400–500 mm^3^). To evaluate the antitumor potency, we treated the immunocompetent mice harboring syngeneic tumors by intravenous injections of PBS, ST8/pSGFP or ST8/pSEndo. Animals in the control groups were uniformly killed by tumors within 2 weeks, whereas the ST8-mediated expression of secretable version of angiogenic inhibitors slowed a robust anti-tumoral effect and sometimes shrunk the established tumors, with all animals were alive during the observed time after bacterial injection (Fig. 5a). In addition, immunohistochemical detection of proliferating marker Ki67 also showed that the proliferating cells were largely reduced in the ST8/pSEndo group compared with ST8/pSGFP (Fig. 5b). Thus, ST8 has a propensity to multiply preferentially in solid tumors and consequently retards tumor growth.

During the treatments, the animals in the bacterially treated groups showed a transient weight loss. However, the observed weight loss was totally reversible after several days post injections **(**Additional file 1: Figure S2). ST8/pSEndo infection did not result in the spleen swelling **(**Additional file 1: Figure S3). Gross morphological examination of lung, liver and spleen showed that there were no detectable abnormal nodules and apoptotic cell death in the selected organ (Fig. 5c). Collectively, these experiments showed that ST8/pSEndo are well tolerated at the dosage selected and require 2–3 weeks for full clearance. Taken together, treatment with ST8/pSEndo reduced the number of tumor-associated blood vessels, and tumor size at later disease stages, without detectable systemic toxicity.

## Discussion

To circumvent the problem of targeting genetically unstable tumor cells, another potential direction is to target stable, proliferating endothelial cells in the tumor vasculature to inhibit angiogenesis (Weis and Cheresh 2011). It has been widely recognized that angiogenesis is a critical process required by solid tumors to support their growth. Here, we engineered a *Salmonella* strain ST8 to selectively grow within tumors, where they cause lysis of tumor cells and secrete the biologically active angiogenic inhibitors in situ at high regional concentration, thereby achieving maximal inhibiting effects while sparing system cytotoxity.

Previous studies have shown that the localization of antitumor proteins within live bacterial strains may play a key role in the therapeutic effects (Bernardes et al. 2013; Chen et al. 2011). Therefore, to elicit maximal antitumor responses, bacterial-mediated secretion of therapeutic factors without the requirement of bacterial lysis have been investigated in this study. Furthermore, to maintain of high protein production capacity in vivo, ST8 strain bearing high-copy number plasmids encoding endostatin gene fused with location signal were used. Therefore, just a single dose of administration is needed instead of daily administration of the proteins. As a result, the cost and time could be saved and the variation of therapeutic protein levels in the circulation could be avoided.

As has been proven by several laboratories, *Salmonella* alone could induce modest antitumor effects in animal models although the mechanisms responsible for this are not yet fully understood (Hiroshima et al. 2013; Murakami et al. 2015). The combination of bacteria with angiogenic inhibitors may elicit a strong synergistic effect and directly contribute to tumor destruction through the release of nitric oxide (Griffon et al. 1998), protease (Le Negrate et al. 2008), pore-forming agents (Shi et al. 2016) and therapeutic factors. This dual mode of action through direct cell infection and extracellular secretion has not been described before and opens up new strategies for eradicating cancer effectively through this kind of combination. Lastly, it should be noted this therapeutic platform is not necessarily restricted to cancer. It is suitable for indirectly acting therapeutic strategies such as anti-angiogenesis and immune therapy.

## Additional files

10.1186/s13568-016-0226-8 Additional tables and figures.

## Abbreviations

CFU: colony forming units
CM: conditioned medium
DAB: 3,3-diaminobenzidine
DAP: diaminopimelic acid
HUVEC: human umbilical vein endothelial cells
H&E: hematoxylin and eosin
LB: Luria broth
MOI: multiplicity of infection
SD: standard deviation
T3SS: type III secretion system
