# Supplementary material for: Angiogenic inhibitors delivered by the type III secretion system of tumor-targeting Salmonella typhimurium safely shrink tumors in mice
Source: AMB Express. 2016 Aug 24;6(1):56. doi: 10.1186/s13568-016-0226-8 (PMC4996802; doi:10.1186/s13568-016-0226-8)
Supplement: Supplementary file 1 — 10.1186/s13568-016-0226-8 Additional tables and figures. [file 13568_2016_226_MOESM1_ESM.pdf]

Journal name: AMB express

Manuscript Title: Angiogenic inhibitors delivered by the type III secretion system of tumor-targeting *Salmonella typhimurium* safely shrink tumors in mice

Author affiliation

Lei Shi[1,2], Bin Yu [1,2,3], Chun-Hui Cai[4,5], Jian-Dong Huang [1,2,6,*]

[1] *School of Biomedical Sciences; the University of Hong Kong; Pokfulam, Hong Kong;*

[2] *Shenzhen Institute of Research and Innovation; the University of Hong Kong; Shenzhen, PR China;*

[3] *Key Laboratory of Optoelectronic Devices and Systems of Ministry of Education and Guangdong Province; College of Optoelectronic Engineering; Shenzhen University; Shenzhen, PR China;*

[4] *Department of Obstetrics and Gynaecology; the University of Hong Kong; Pokfulam, Hong Kong;*

[5] *Advanced Institute of Translational Medicine, Tongji University School of Medicine; Shanghai, PR China;*

[6] *The Centre for Synthetic Biology Engineering Research; Shenzhen Institutes of Advanced Technology; Shenzhen, PR China.*

Corresponding author

* Correspondence and requests for materials should be addressed to Jian-Dong Huang.

Location: L3-72, Laboratory Block, Faculty of Medicine, the University of Hong Kong, 21 Sassoon Road, Hong Kong.

Office Phone: +852 3917 2810

Fax number: +852 2855 1254

E-mail: jdhuang@hku.hk

Supplementary materials

Table S1. Bacterial strains and plasmids used in this study

	Relevant genotype
SL7207	*S. typhimurium* 2337-65 derivative *his*G46,DEL407 [aroA::Tn10(Tc-s)]; wild type
ST4	SL7207 *⊿gmd:: Plac-T7 RNAP; ⊿asd::PsseA-hlyA; ⊿htrA::Cm-PpepT-asd-sodA; ⊿infA::Ptet-tetR*
ST7	SL7207 *⊿gmd::Plac-T7 RNAP; ⊿asd::PsseA-hlyA; ⊿htrA::Cm-Pcons(BBa_J23109)-asd-PsodA; ⊿infA::Ptet-tetR*
ST8	SL7207 *⊿gmd::Plac-T7 RNAP; ⊿asd::PsseA-hlyA; ⊿htrA:: PpepT-asd-PsodA; ⊿infA::Ptet-tetR*
pcDNA-infA	ApR; pcDNA3.1 (+) derivative with *an infA* locus from *E.coli* MG1655 strain
pET32a-infA	ApR; pET32a (+) derivative with *an infA* locus from *E.coli* MG1655 strain
pSEndo	ApR; pET32a-infA derivative with mouse *Endostatin* cDNA fused with a N-terminal FLAG tag and a N-terminal *SopA* sequence
pSGFP	ApR; pET32a-infA derivative with EGFP fused with Flag tag and a N-terminal *SopA* sequence

Table S2. Details of oligonuclotides used in this study.

Name	Sequence	Purposes
stuI-MG1655-infA-f	AGGCCTTTTACTTATTTACAGAACT T	pcDNA3.1(+)-infA pET32a (+)-infA
BssHII-MG1655-infA -r	GCGCGCATAAAAAGGCCGGTTAAA CC	pcDNA3.1(+)-infA pET32a (+)-infA
NcoI-FLAG-SopA-F	GCGCCATGGATTACAAGGATGACG ACGATAAGATATCATCAGGCGCAAT	pSEndo pSGFP
NdeI-SopA-R	GCGCATATGCTTGCCTGCATTATTTG TAT	pSEndo pSGFP
HindIII-PstI-rmEnd-r	AAGCTTCTGCAGTTATTTGGAGAAA GAGGTCATG	pSEndo

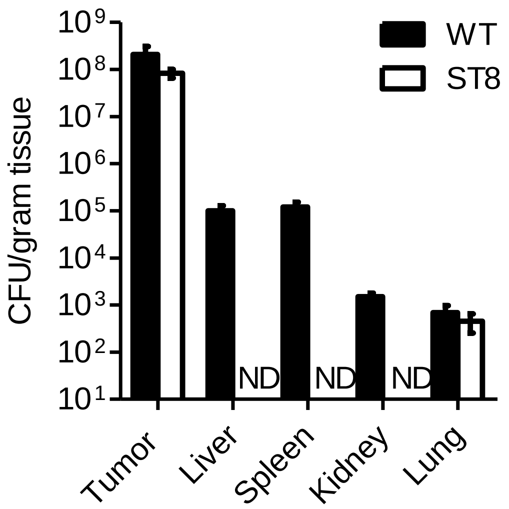

Figure S1. CFU tests of wild type *Salmonella* SL7207 and ST8 inside tumors of 4T1 breast tumor bearing mice following intravenous injection. Different organs were collected at 14 days after bacterial infection, homogenized in PBS and plated onto agar plates to determine the counts of bacteria, respectively. Values are expressed as mean ± SD, n=3.ND, not detected (CFU=0).

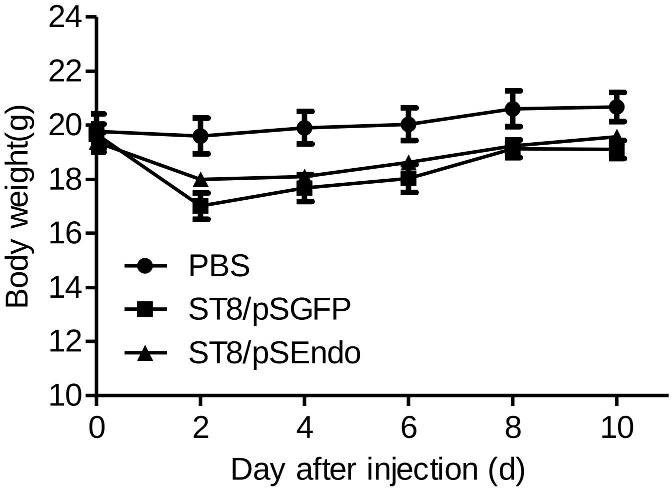

Figure S2. Body weight of mice received with PBS, ST8/pSGFP or ST8/pSEndo treatments.

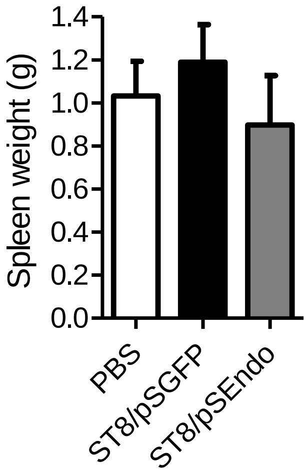

Figure S3. Spleen weights in PBS, ST8/pSGFP and ST8/pSEndo treated groups indicated no significant difference. (PBS vs ST8/pSEndo, P=0.3002).
